# Management of Idiots

**Published:** 1844-10-19

**Authors:** 


#### MANAGEMENT OF IDIOTS.

M. Ed. Seguin has just written a work, in which he makes the following important remarks:

"By gymnastic exercises, properly and suitably regulated, the muscular system is strengthened; by mechanical excitation, the voluntary muscles of the limbs, the trunk and face, are exercised; by the dumb bells and the balancing pole, both valves of the body are regulated in their forces, which creates an uprightness in standing, walking, &c.; by the exercise of the senses, the subject is placed in precise and rapid communication with himself and the external world; he is disposed to intellectual life by the study of notions, and notions lead to re-connected ideas; by talking, by reading, and writing, he is made to enter upon abstract questions, when numbers and morals give him the feeling of relations which he should establish with his equals. Many children abandoned as idiots, may be thus far conducted; but no doubt a certain number of them can never go beyond the limits which separate notions from ideas, or connected from abstract ideas. There is still a small number in whom education can only modify the most repulsive habits, especially those in whom idiotcy is complicated with epilepsy, paralysis, rachitis, &c. For the same reason that there are incurable diseases, we must also recognize cases of idiotcy resisting almost all possible means of education. But that is no reason for abandoning without exception all idiots to the deplorable state in which we find them. The time is come to do something for these poor creatures, analogous to that which is practiced in the numerous schools for the deaf and dumb."

These are sound principles, and experience has proved that they may be successfully put in practice. It is very desirable that they should be carried out upon a large scale, and meet with approbation and support from the general public.—*Gaz. Med. de Paris.*

